# *Announcement: *Implementation of the Vaccine Adverse Event Reporting System 2.0 Reporting Form

**DOI:** 10.15585/mmwr.mm6627a5

**Published:** 2017-07-14

The Vaccine Adverse Event Reporting System (VAERS), co-managed by CDC and the Food and Drug Administration (FDA), is the national postmarketing safety monitoring system that accepts reports about adverse events that occur after administration of U.S.-licensed vaccines (*1*,*2*). On June 30, 2017, CDC and FDA implemented a revised reporting form and a new process for submitting reports to VAERS. Persons reporting adverse events are now able to use the VAERS 2.0 online reporting tool to submit reports directly online; alternatively, they may download and complete the writable and savable VAERS 2.0 form and submit it using an electronic document upload feature.

Transition to the VAERS 2.0 form is expected to be completed by the end of December 2017. Accommodations will be made for persons unable to submit reports electronically. The revised VAERS reporting form and system is intended for health care professionals, patients, parents, guardians, caregivers and other nonmanufacturers. Vaccine manufacturers will submit reports to VAERS by a separate process through the FDA Electronic Submissions Gateway (*3*). Instructions for reporting to VAERS are available at https://vaers.hhs.gov/reportevent.html. Additional assistance is available via email at info@vaers.org or by telephone at 1-800-822-7967.
